# Fitterfly Diabetes CGM Digital Therapeutics Program for Glycemic Control and Weight Management in People With Type 2 Diabetes Mellitus: Real-world Effectiveness Evaluation

**DOI:** 10.2196/43292

**Published:** 2023-05-03

**Authors:** Shilpa Joshi, Ritika Verma, Tejal Lathia, Chitra Selvan, Snehal Tanna, Amit Saraf, Mangesh Tiwaskar, Alok Modi, Sanjay Kalra, Vasudevarao K, Manoj Chitale, Forum Malde, Mohammed Abdul Khader, Arbinder Kumar Singal

**Affiliations:** 1 Department of Metabolic Nutrition Fitterfly HealthTech Pvt Ltd Navi Mumbai India; 2 Department of Scientific writing and Research Fitterfly HealthTech Pvt Ltd Navi Mumbai India; 3 Department of Endocrinology and Diabetology Apollo Hospitals Navi Mumbai India; 4 Department of Endocrinology and Diabetology MS Ramaiah Memorial Hospital Bangalore India; 5 Department of Internal Medicine Jupiter Hospital Thane India; 6 Department of Diabetology Shilpa Medical Research Center Mumbai India; 7 Department of General Medicine Kevalya Hospital Thane India; 8 Department of Endocrinology and Diabetology Bharti Research Institute of Diabetes and Endocrinology Haryana India; 9 Department of Endocrinology and Diabetology Hridayam Diabetes World Thane India; 10 Department of General Medicine Shree Clinic Nashik India; 11 Office of Chief Executive Officer Fitterfly HealthTech Pvt Ltd Navi Mumbai India

**Keywords:** digital therapeutics, glycemic control, continuous glucose monitoring, monitoring, glucose, diabetes, type 2 diabetes, decision-making, model, glycemic, effectiveness, mobile application, application, engagement

## Abstract

**Background:**

Digital therapeutic platforms facilitate health care through patient-centered strategies based on multidisciplinary teams and shared decision-making. Such platforms can be used for developing a dynamic model of diabetes care delivery, which can help in improving glycemic control by promoting long-term behavior changes in people with diabetes.

**Objective:**

This study aims to evaluate the real-world effectiveness of the Fitterfly Diabetes CGM digital therapeutics program for improving glycemic control in people with type 2 diabetes mellitus (T2DM) after the completion of 90 days in the program.

**Methods:**

We analyzed deidentified data of 109 participants in the Fitterfly Diabetes CGM program. This program was delivered through the Fitterfly mobile app coupled with continuous glucose monitoring (CGM) technology. This program consists of 3 phases: the first phase is observation, wherein the patient’s CGM readings are observed for 7 days (week 1); the second phase is the intervention; and the third phase aims at sustaining the lifestyle modification introduced during the second phase. The primary outcome of our study was the change in the participants’ hemoglobin A_1c_ (HbA_1c_) levels after program completion. We also evaluated the changes in participant weight and BMI after the program, changes in the CGM metrics in the initial 2 weeks of the program, and the effects of participant engagement in the program on improving their clinical outcomes.

**Results:**

At the end of the 90 days of the program, the mean HbA_1c_ levels, weight, and BMI of the participants were significantly reduced by 1.2% (SD 1.6%), 2.05 (SD 2.84) kg, and 0.74 (SD 1.02) kg/m^2^ from baseline values of 8.4% (SD 1.7%), 74.45 (SD 14.96) kg, and 27.44 (SD 4.69) kg/m^2^ in week 1, respectively (*P*<.001). The average blood glucose levels and time above range values showed a significant mean reduction by 16.44 (SD 32.05) mg/dL and 8.7% (SD 17.1%) in week 2 from week 1 baseline values of 152.90 (SD 51.63) mg/dL and 36.7% (SD 28.4%), respectively (*P*<.001 for both). Time in range values significantly improved by 7.1% (SD 16.7%) from a baseline value of 57.5% (SD 25%) in week 1 (*P*<.001). Of all the participants, 46.9% (50/109) showed HbA_1c_ reduction ≥1% and 38.5% (42/109) showed weight loss ≥4%. The average number of times the mobile app was opened by each participant during the program was 108.80 (SD 127.91) times.

**Conclusions:**

Our study shows that participants in the Fitterfly Diabetes CGM program showed a significant improvement in their glycemic control and reduction in weight and BMI. They also showed a high level of engagement with the program. Weight reduction was significantly associated with higher participant engagement with the program. Thus, this digital therapeutic program can be considered as an effective tool for improving glycemic control in people with T2DM.

## Introduction

### Background

Diabetes mellitus affects more than 536 million adults globally, and the incidence is projected to rise to 783 million by 2045 [1]. Type 2 diabetes mellitus (T2DM) accounts for the majority of cases with diabetes, and the trend is expected to be similar in 2045. Many studies on people with T2DM have shown that the degree of hyperglycemia is associated with the risk of microvascular complications [2,3], neuropathy [4], stroke [2], myocardial infarction [5], macrovascular mortality [6], and all-cause mortality [5,7]. Glycemic variability is also associated with the development of complications in diabetes [8,9].

Diabetes care delivery is currently facing challenges such as skewed patient-to-physician ratios [10,11], inadequate diabetes self-management education and support [12], fragmented care across individual health care providers [13], inadequate resources for glucose monitoring [14], and lack of awareness about dietary management practices that often lead to poor glycemic control [15]. High-quality diabetes care depends on the adoption of comprehensible lifestyle management techniques, which include diabetes self-management education and support, nutritional therapy, physical activity, regular glucose monitoring, psychological care, and smoking cessation [16]. Patients with diabetes are challenged with a huge burden of self-care, including regular glucose monitoring, dietary management, meal tracking, and physical activity on a daily basis, which has been shown to result in lower treatment adherence and psychological burnout [17]. Regular self-care can become exhausting, and education and support from experts can help in reducing the onset and progression of diabetes complications [18]. Along with this, a personalized treatment approach can help people with diabetes reach their glycemic targets [19] and reduce the risk of T2DM-related complications [14]. Thus, there is an urgent need for a dynamic model of diabetes care delivery using patient-centered strategies based on multidisciplinary teams and shared decision-making, which can help in improving glycemic control by promoting long-term behavior changes in people with diabetes [20]. Digital therapeutic platforms provide evidence-based therapeutic interventions with the use of high-quality software to prevent, manage, or treat a medical disorder or disease [21]. Digital therapeutic platforms have been shown to provide diabetes care independently or in concert with medications, devices, or other therapies to optimize patient care and health outcomes [22].

Fitterfly Diabetes CGM is a 90-day digital therapeutics program that provides personalized lifestyle management support for people with T2DM. This program consists of the initial application of the continuous glucose monitoring (CGM) sensor on the patient and the concurrent detailed profiling of the patient’s glucose readings and fitness and stress assessments by a multidisciplinary care team of experts comprising nutritionists, physiotherapists, and psychologists. In this program, CGM readings are correlated with a vast set of input data (food logs, activity logs, symptoms, medication, sleep, and stress) from the mobile app. Artificial intelligence and machine learning predictive models are used to predict the personalized glycemic response of the individuals, by assessing the impact of food, activity, medication, sleep, symptoms, and stress on blood glucose excursions.

In a previous study performed on 64 participants with T2DM enrolled in the Fitterfly Diabetes CGM program (formerly known as Diabefly-Pro; Fitterfly Healthtech Pvt Ltd), CGM data were analyzed only in the initial 14 days of the program. Participants followed their usual lifestyle in week 1 but a modified lifestyle plan in week 2. In week 2, the mean blood glucose level was significantly reduced by 17.5 mg/dL (*P*<.001) and the time in range (TIR) and the glucose management indicator significantly improved by 4.5% and 0.4%, respectively (*P*<.001), while the time above range (TAR) reduced significantly by 11% (*P*<.001). The changes in the CGM metrics in that study indicated that the Fitterfly Diabetes CGM program significantly improved glycemic control in a short duration [23]. However, the extent of improvement in glycemic control after the completion of the program at 90 days was not studied. Therefore, our study aims to analyze the deidentified data of 109 participants with T2DM in the Fitterfly Diabetes CGM program for assessing its real-world effectiveness in improving glycemic control in 90 days.

### Objective

The Fitterfly Diabetes CGM program aims at providing personalized lifestyle management with the help of the CGM sensor (FreeStyle Libre Pro, Abbott Diabetes Care). The primary outcome of our study was to analyze the changes in the hemoglobin A_1c_ (HbA_1c_) levels after the completion of the program as compared to those in the baseline. We also focused on evaluating the short-term effects of a modified lifestyle plan by using the CGM metrics of the participants, changes in their weight and BMI after program completion, and the effects of participant engagement in the program on their clinical outcomes.

## Methods

### Study Design

This study is based on the analysis of the deidentified data of participants enrolled in the Fitterfly Diabetes CGM program. Participants were recruited through direct referrals by the treating physician or via a social media campaign offering an app-based diabetes management program. Participants were enrolled in the program only after the screening process, which was based on the following inclusion and exclusion criteria. The inclusion criteria for the participants were (1) diagnosis of T2DM with HbA_1c_ levels >6.5%, (2) age ≥18 years at the time of enrollment, (3) having a smartphone and willing to utilize the mobile app, and (4) having a minimum level of literacy to read and understand the English language. The exclusion criteria for the participants were (1) presence of any physical, cognitive, and psychiatric impairments, which can affect their ability to follow dietary regimens or physical exercise, (2) presence of severe complications (eg, end-stage chronic kidney failure, chronic liver disease), (3) history of unstable angina pectoris or stroke within the past 6 months, and (4) history of surgical procedures, which can affect the ability to follow a dietary regimen. All participants signed informed consent for the use of data for research purposes. Refusal to sign the informed consent form did not affect their participation in the program and the quality of care.

### Ethical Considerations

This study involves secondary analysis of deidentified data, and no investigational product or procedures were used in this study. Thus, no ethics clearance was obtained for this study. All participants were provided care as per normal clinical standards, and there was no change in their treatment from the usual customary care. All participants signed the informed consent permitting the collection of primary data for the secondary analysis of deidentified data for research purposes and publications. Our study guarantees the protection of privacy and confidentiality of participants by ensuring that the study data are deidentified. Participants were not provided any compensation for study participation.

### Program Details

**Figure 1:**
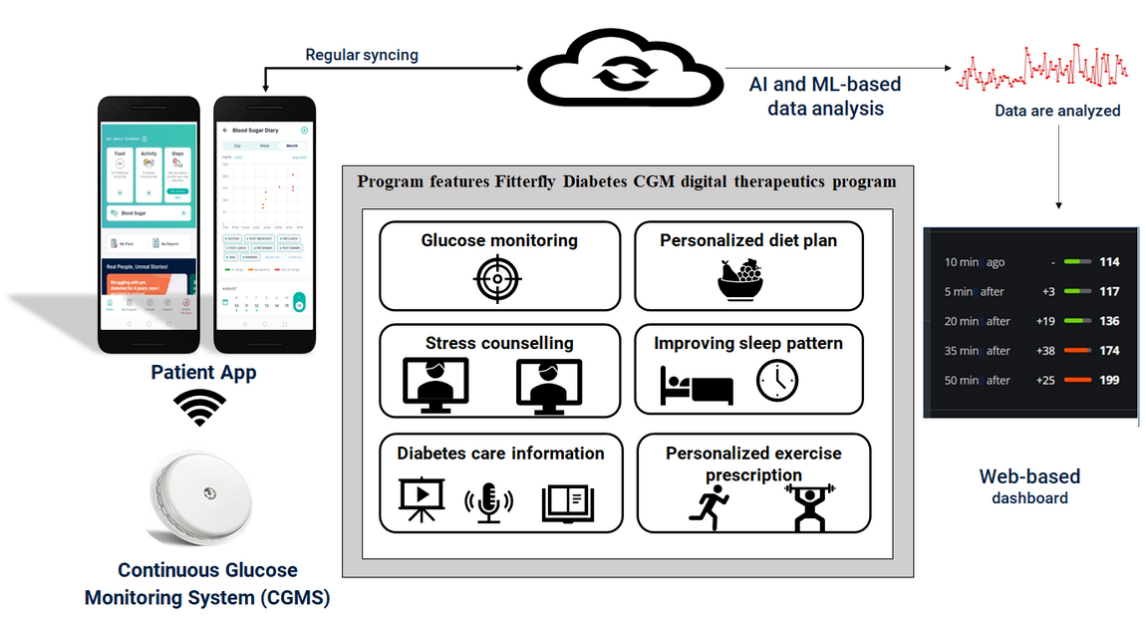
Process flow of the Fitterfly diabetes CGM digital therapeutics program. AI: artificial intelligence; CGM: continuous glucose monitoring; ML: machine learning.

Figure 1 shows the major components of the Fitterfly Diabetes CGM program. The diabetes management program was delivered through a Fitterfly mobile app coupled with CGM technology. Personalized guidance was provided based on data insights on the blood glucose levels through CGM monitoring and the data entered by participants through the mobile app, including meals items, duration and type of physical activity performed, normal lifestyle habits, diabetes distress score, anthropometric parameters, symptoms, sleep quality, medication usage, and laboratory reports. The Fitterfly Diabetes CGM program uses machine learning and artificial intelligence models to integrate and correlate the data collected from the CGM device and the mobile app to create a personalized lifestyle plan based on an individual’s glycemic response.

The key differentiating features of the Fitterfly Diabetes CGM program are as follows:

1. Mobile app software: The Fitterfly mobile app software helps in logging of anthropometric and other data (HbA_1c_ levels; meals; physical activity; anthropometric data such as height, waist circumference, and weight) along with ensuring regular access to diabetes education content (lessons, quizzes, articles, blogs) based on evidence-based guidelines. Regular reports (nutrition, physical fitness, and psychological well-being assessment) are also created and shared via the mobile app with the participants during the 90 days of the program.
2. Food database: The in-house food database access was provided through the mobile app. The food database provides dietary information such as calories, macronutrients (protein, fat, and carbohydrate), micronutrients (calcium, phosphorus, iron, sodium, potassium, zinc, magnesium), omega-3 fats, and total fiber in various meal items (17,187 recipes, 13,866 packed foods, and 152 cuisines). The food database was created from authentic sources such as the Indian Food Composition tables 2017 and the National Institute of Nutrition, India.
3. Virtual access to nutritionists: Meal logs created by the participants were reviewed by nutritionists, and a personalized diet plan was created and shared regularly with each participant based on their personalized glycemic response. The team made regular calls to assess the goals achieved by the participants and to keep them on track.
4. Virtual access to physiotherapists: Personalized exercise plans were created for participants based on a videocall-based physical fitness assessment in the initial phase of the program by trained physiotherapists. The personalized exercise prescription is based on physical fitness, pain complaints of participants, and physical activity readiness level. Regular calls by physiotherapists were provided to assess the goals achieved by participants and to keep them on track. The participants underwent videocall-based primary fitness assessments using the 6-minute walk test, 1-minute push-up test, wall sit test, 1-minute sit-up test, and V-sit and reach test for the analysis of cardiorespiratory fitness, upper body strength, lower body strength, core strength, and flexibility, respectively, under the supervision of trained physiotherapists at the beginning and end of the program.
5. Virtual access to psychologists: The main objectives of psychological care were to enhance motivation, positivity, and optimism; improve sleep quality; manage stress; and learn self-control techniques for better adherence to the program and for providing care for diabetes-related distress in participants. The participants are provided with video and activity cards via the mobile app. Regular calls were performed by the psychologists to assess the goals achieved by the participants and to keep them on track. Psychological well-being assessment was conducted using questionnaire-based scales such as Diabetes Distress Scale, Motivation and Attitude toward Changing Health, and Pittsburgh Sleep Quality Index, which were administered using the mobile app at the beginning and end of the program.
6. Remote health coaching: Remote health coaches help the participants with their queries or problems faced by participants through messages and calls.
7. Dashboard: Web-based dashboards helped in integrating all the information and ensured easy access of the data to remote health coaches and experts (nutritionists, physiotherapists, and psychologists) during the program.

The Fitterfly Diabetes CGM program consists of 3 phases. The first phase is the observation phase, wherein the CGM readings of the participants are monitored for 7 days (week 1) based on their normal lifestyles (daily activity, meals, stress, and sleep quality). The second phase is the intervention phase, during which nutritionists and physiotherapists provide every participant with a diet and exercise plan, respectively, based on their personalized glycemic response data collected from the CGM monitoring sheet. The participants were instructed to follow the modified diet and exercise plan and were monitored again for the next 7 days (week 2) through the CGM device. Feedback regarding stress management and sleep quality was also provided. The third phase of the program aims at sustaining the lifestyle modification introduced during the second phase of the program while including regular feedback and support from health coaches to build lifelong lifestyle changes for better management of diabetes.

### Data Collection

The primary outcome of this study was to evaluate the change in the HbA_1c_ levels. The secondary outcomes of this study were reductions in weight and BMI, psychological well-being, and physical fitness. Before the start of the program, all the participants downloaded the Fitterfly mobile app and trained personnel applied the CGM sensor on the participant. CGM readings were collected on day 7 and day 14 of the program by trained personnel during their personal visits to participants. All the participants completed a profiling questionnaire, which helped in personalizing the participant’s experience. The profiling questionnaires were administered using the mobile app.

### Statistical Analysis

Statistical analysis was performed using the R software (version 4.0.3, R Core Team and the R Foundation for Statistical Computing). Continuous data were expressed as mean (SD) and median (IQR). Categorical data were represented as number (%). Shapiro-Wilk test was used for normality assessment of data. Wilcoxon signed-rank test was performed for the evaluation of outcomes before and after the program, with *P*≤.05 considered as statistically significant. The correlation between various factors was studied using Pearson test for parametric data and Spearman rank test for nonparametric data.

## Results

### Baseline Characteristics of the Participants

We obtained complete CGM readings from 355 participants at the beginning and after 14 days of the program start date. Complete readings of HbA_1c_ levels before and after the program were provided by 112 participants. Complete weight readings were provided by 109 participants. Table 1 shows the baseline characteristics of 109 participants with T2DM who participated in the Fitterfly Diabetes CGM program. The mean age of the participants was 48.90 (SD 12.70) years, with an average duration of diabetes history of 5.37 (SD 8.37) years. Female participants comprised 55.9% (61/109) of this study population. The mean weight and BMI at the baseline were 74.45 (SD 14.96) kg and 27.44 (SD 4.69) kg/m^2^, respectively. Apart from being diagnosed with diabetes, 53.2% (58/109) of the participants had other health comorbidities. The analysis of participants’ usage of antidiabetic agents while on the program showed that 13.8% (15/109) of the participants were using insulin, 40.4% (44/109) were using oral hypoglycemic agents, 20.2% (22/109) were using both insulin with oral hypoglycemic agents, and 25.7% (28/109) were not using any form of antidiabetic medications. Of all the participants, 47.7% (52/109), 32.1% (35/109), 32.1% (35/109), 13.8% (15/109), 8.3% (9/109), and 1.8% (2/109) were using biguanides, sulfonylureas, dipeptidyl peptidase-4 inhibitors, sodium-glucose co-transporter-2 inhibitors, α-glucosidase inhibitors, and thiazolidinediones, respectively.

**Table 1:** Baseline characteristics of the study participants (N=109).

Parameters	Values
Gender (female), n (%)	61 (55.9)
Age (years), mean (SD)	48.90 (12.70)
Duration of diabetes (years), mean (SD)	5.37 (8.37)
BMI (kg/m^2^), mean (SD)	27.44 (4.69)
Weight (kg), mean (SD)	74.45 (14.96)
Insulin, n (%)	15 (13.8)
Oral hypoglycemic agents, n (%)	44 (40.4)
Insulin and oral hypoglycemic agents, n (%)	22 (20.2)
Biguanides, n (%)	52 (47.7)
Sulfonylurea, n (%)	35 (32.1)
Dipeptidyl peptidase-4 inhibitors, n (%)	35 (32.1)
Sodium glucose co-transporter-2 inhibitors, n (%)	15 (13.8)
α-Glucosidase inhibitors, n (%)	9 (8.3)
Thiazolidinediones, n (%)	2 (1.8)
Other/nonspecified medication, n (%)	2 (1.8)
Comorbid conditions present, n (%)	58 (53.2)

### Changes in CGM Metrics After the Initiation of a Modified Lifestyle Plan

Personalized feedback was provided for all the participants based on the analysis of CGM data in the first week of the program. From the second week to the end of the program, the participants followed modified lifestyle prescriptions. CGM sensor data for the second week of the program were compared with those in the first week of the program to understand the immediate change in the glycemic parameters after the introduction of the modified lifestyle plan. The average blood glucose levels of the participants showed a mean reduction by 16.44 (SD 32.05) mg/dL from 152.9 (SD 51.63) mg/dL in week 1 to 136.50 (44.26) mg/dL in week 2. TIR improved by 7.1% (SD 16.7%) from week 1—from a baseline value of 57.5% (SD 25%) to 64.6% (SD 26%) (*P*<.001). TAR significantly reduced by 8.7% (SD 17.1%) from week 1—from a baseline value of 36.7% (SD 28.4%) to 28.1% (SD 28.1%) (*P*<.001). No significant increase in time below range (TBR) was observed between week 1 and week 2 (*P*=.86) (Table 2).

**Table 2:** Summary of the parameters in the participants before and after the Fitterfly Diabetes continuous glucose monitoring intervention program.

Parameters	Preintervention, mean (SD), median (IQR)	Postintervention, mean (SD), median (IQR)	Change in parameters, mean (SD), median (IQR)	*P* value^a^
Hemoglobin A_1c_ (%)	8.4 (1.7), 8.1 (7.0 to 9.1)	7.2 (1.4), 7.1 (6.4 to 7.8)	–1.2 (1.6), –0.9 (–1.9 to –0.3)	<.001
Weight (kg)	74.45 (14.96), 73.0 (64.50 to 82.50)	72.40 (13.92), 71.0 (64.0 to 80.0)	–2.05 (2.84), –1.40 (–4.0 to 0)	<.001
BMI (kg/m^2^)	27.44 (4.69), 26.50 (23.85 to 30.35)	26.70 (4.41), 25.98 (23.43 to 29.53)	–0.74 (1.02), –0.55 (–1.41 to 0)	<.001
ABG^b^ (mg/dL)	152.90 (51.63), 139.00 (120.0 to 171.50)	136.50 (44.26), 125.00 (108.0 to 155.50)	–16.44 (32.05), –10.00 (–22.50 to –1.50)	<.001
TIR^c^ (%)	57.5 (25.0), 61.0 (45.1 to 75.0)	64.6 (26.0), 72.0 (48.0 to 83.5)	7.1 (16.7), 6.0 (–0.2 to 16.1)	<.001
TAR^d^ (%)	36.7 (28.4), 32.7 (13.8 to 51.7)	28.1 (28.1), 16.9 (6.3 to 41.2)	–8.7 (17.1), –5.2 (–16.8 to 0.0)	<.001
TBR^e^ (%)	6.0 (11.8), 1.1 (0.0 to 5.6)	7.5 (13.3), 0.9 (0.0 to 8.7)	1.5 (11.2), 0 (–1.3 to 1.0)	.86

^a^Wilcoxon signed-rank test.

^b^ABG: average blood glucose.

^c^TIR: time in range.

^d^TAR: time above range.

^e^TBR: time below range.

### Changes in HbA_1c_ Levels After Program Completion

**Figure 2:**
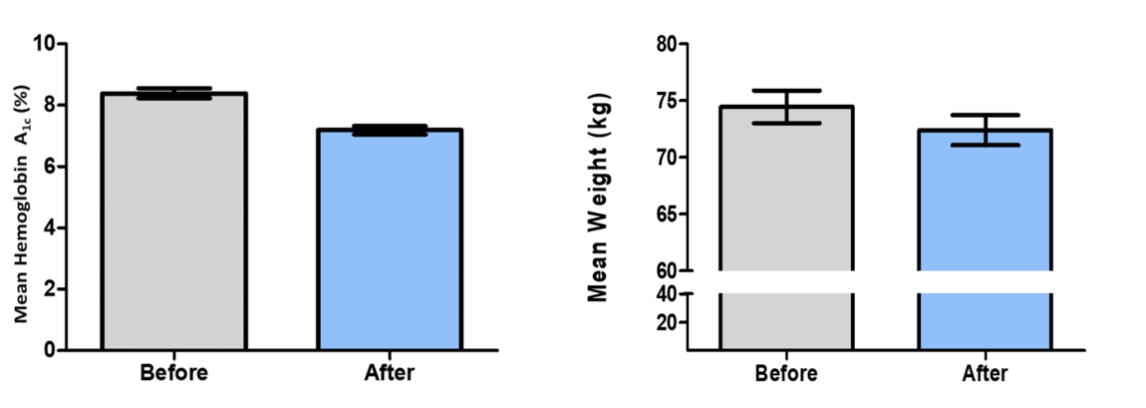
Changes in (A) hemoglobin A1c level and (B) weight before and after the program.

A significant mean reduction in HbA_1c_ levels by 1.2% (SD 1.6%) (*P*<.001) was observed in all the participants—from a baseline mean of 8.4% (SD 1.7%) to 7.2% (SD 1.4%) after the program (Figure 2A). Of all the participants, 85.3% (93/109) showed a reduction in HbA_1c_ levels after the program, with 43.1% (47/109) reaching the recommended target of HbA_1c_<7%. Approximately 46.9% (50/109) of the participants showed HbA_1c_ reduction ≥1%, and 86.2% (94/109) of the participants showed reduction in HbA_1c_ levels. Participants with baseline HbA_1c_<7%, 7%-9%, and >9% showed an average HbA_1c_ reduction by 0.4% (SD 0.7%) (*P*=.008), 0.9% (SD 1.5%) (*P*<.001), and 2.6% (SD 1.7%) (*P*<.001), respectively.

### Changes in Weight and BMI After Program Completion

The participants showed a significant mean weight reduction by 2.05 (SD 2.84) kg from a preprogram mean weight of 74.45 (SD 14.96) kg to 72.40 (SD 13.92) kg after the program (*P*<.001, Figure 2B). Weight reduction was observed in 65.1% (71/109) of the participants, with 38.5% (42/109) having weight loss of ≥4%. The mean BMI reduced significantly by 0.74 (SD 1.02) kg/m^2^ among all the participants—from a mean baseline BMI of 27.44 (SD 4.69) kg/m^2^ to 26.70 (SD 4.41) kg/m^2^ (*P*<.001).

### Changes in Glycemic Control With Different Antidiabetic Medications

TIR improvement and HbA_1c_ reduction were further analyzed in all the participants based on their uptake of antidiabetic medications for glycemic control, such as insulin, oral hypoglycemic agents, oral hypoglycemic agents with insulin, and no medications. Participants who were not using any antidiabetic medications were dependent solely on the lifestyle modifications for glycemic control. Similar levels of improvement in TIR were observed in the groups receiving oral hypoglycemic agents, insulin, oral hypoglycemic agents with insulin, and no medications, with an average TIR improvement of 6.6% (SD 17.9%), 5.7% (SD 24.6%), 8.3% (SD 14%), and 7.9% (SD 12%), respectively (*P*=.94). Similarly, no significant difference in the level of HbA_1c_ reduction was observed between these 4 groups, with a mean HbA_1c_ reduction of 1.1% (SD 1.4%), 1.5% (SD 1.3%), 1.4% (SD 2.1%), and 1.1% (SD 1.7%), respectively (*P*=.61).

### Program Engagement and Glycemic Control

Participant engagement with the mobile app was studied during the entire 90 days of the program. The average number of times the mobile app was opened by each participant was 108.80 (SD 127.91) times (1.2 logins per day per participant). The average call duration per participant was 1.35 (SD 1.10) hours in 90 days. The average number of meal entries, physical activity entries, and number of body composition entries by each participant during the entire 90 days was 242.18 (SD 244.75), 27.65 (SD 42.01), and 29.35 (SD 29.71), respectively. Reduction in participant weight after the program showed significant correlation with the number of times the mobile app was opened (ρ=0.191; *P*=.04), call duration (ρ=0.221; *P*=.02), meal entry count (ρ=0.246; *P*=.01), activity entry count (ρ=0.315; *P*=.01), and number of body composition entries (ρ=0.227; *P*=.01).

## Discussion

### Major Findings

Our study discusses the real-world effectiveness of the Fitterfly Diabetes CGM digital therapeutics program for improving glycemic control in people with T2DM after 90 days of program participation. The analysis of deidentified data of 109 participants with T2DM showed the short-term changes in glycemic control after the introduction of a modified lifestyle plan, with improvement in TIR by 7.1% along with a reduction in TAR by 8.7% and no significant increase in TBR (*P*=.86). After the completion of the program, the participants showed an average reduction in HbA_1c_ level by 1.2%, weight by 2.05 kg, and BMI by 0.74 kg/m^2^. Approximately 85.3% (93/109) of the participants showed a reduction in the HbA_1c_ levels after program completion. No significant variation was observed in the level of HbA_1c_ reduction among participants who were using only lifestyle modification and those who used antidiabetic medications for glycemic control. A significant correlation was observed between participant engagement in the program and weight reduction after the program (*P*=.01 for counts of meal entries, activity entries, and number of body composition entries). CGM-based monitoring helps in assessing intraday and interday glycemic excursions, which can involve episodes of hypoglycemia and postprandial hyperglycemia [24]. Abnormal glycemic excursions have been shown to be associated with both microvascular and macrovascular complications of diabetes [25]. The postprandial glycemic spike and the extent of glycemic excursion can be more adverse than the sustained glycemic response [26]. Dietary intake is the major determinant of blood glucose levels. Significant variations in postmeal glycemic response have been reported in different people eating identical meals [27]. Thus, it is important for people with diabetes to have a clear understanding of their own glycemic response to different meals and their normal lifestyle.

The prediction of personalized glycemic response remains the basis of the Fitterfly Diabetes CGM program, which helps the participants understand their glycemic excursions and receive culturally appropriate techniques by real-time remote expert-based coaching to have their blood glucose levels in the normal range. The CGM data showed significant changes in the glycemic control after 7 days of the introduction of modified lifestyle plans when compared to that during the participants’ usual lifestyle in the first 7 days of the program. Significant reductions in average blood glucose levels and TAR indicated reduction in hyperglycemia. TIR improved by 7.12% (SD 16.73%) over a period of 7 days, indicating improved glycemic control in the participants. No significant change in TBR showed that the modified lifestyle plan did not lead to an increase in hypoglycemia episodes. Earlier studies have shown that reduction in TIR by 10% leads to an increase in the hazard rate for retinopathy progression and microalbuminuria by 64% and 40%, respectively, with every 5% improvement in TIR leading to clear clinical benefit in people with T2DM [28,29]. Thus, the modified lifestyle plan resulted in a clinically significant improvement in glycemic control during week 2 of the program.

At the end of the 90 days in the program, the participants showed an average reduction in HbA_1c_ levels by 1.2%. Clinical studies have shown that a 1% reduction in mean HbA_1c_ levels is associated with a reduction in the risk of myocardial infarction by 14%, death related to diabetes by 21%, and microvascular complications by 37% [30]. Another study conducted in the United States among people with T2DM showed that 1% reduction in HbA_1c_ levels led to 2% reduction in all-cause total health care cost and 13% reduction in diabetes-related total health care cost, leading to per individual annual cost saving of US $429 and US $736, respectively [31]. Of all the participants, 85.3% (93/109) showed a reduction in HbA_1c_ levels after the program, with 46.9% (50/109) showing an HbA_1c_ reduction ≥1%. The program was effective in improving glycemic control in participants with different baseline HbA_1c_ levels, with an average HbA_1c_ reduction by 0.4%, 0.9%, and 2.6% among participants with baseline HbA_1c_ levels <7%, 7%-9%, and >9%, respectively. The participants in our study also showed a significant average reduction in weight by 2.05 kg after 90 days of the program. These results were similar to those reported in studies on other programs using a CGM-based virtual diabetes care for people with T2DM, showing a mean reduction in HbA_1c_ levels by 1.6% (SD 1%) after 4 months [32].

Before the start of the program, 87.2% (95/109) of the participants were in the category of overweight or obesity with an initial BMI ≥23 kg/m^2^ [33]. A significant mean reduction in BMI by 0.74 kg/m^2^ was observed in all the participants (*P*<.001). The reduction in HbA_1c_ levels and improvement in TIR were not significantly different among groups using insulin, oral hypoglycemic agents, insulin with oral hypoglycemic agents, and no medications (*P*=.61 and *P*=.94 for HbA_1c_ and TIR change, respectively). Our findings showed that lifestyle modification can play an important role in improving glycemic control in participants not using any antidiabetic medication and that the Fitterfly Diabetes CGM program played a significant role in improving glycemic control in participants with different medication histories.

Engagement with the program was analyzed to understand the importance of level of participant engagement in the clinical outcomes of the program. High level of participant engagement was observed in the program, with each participant having an average count of 1.2 logins in the mobile app per day and a mean meal entry count per participant being 242 times during the 90 days. Weight reduction showed significant association with higher level of participant engagement in terms of the number of times the mobile app was opened (*P*=.04), call duration with coaches (*P*=.02), meal entry count (*P*=.01), activity entry count (*P*=.01), and the number of times the body composition entry (*P*=.01) was made by the participant. Thus, our study shows that higher engagement with the program led to better outcomes in participants.

The Fitterfly Diabetes CGM program can be highly beneficial to people with T2DM for improving glycemic control. Virtual access to experts from multidisciplinary fields can help in improving diabetes care substantially, especially in low-income countries like India. The application of machine learning and artificial intelligence technology for the prediction of personalized glycemic response with the help of CGM is an emerging technology in India. Further, the program being focused on HbA_1c_ levels as the outcome for lifestyle modification can help in achieving goals in a measured way. The CGM-based analysis in the initial phase of the program helped in providing a personalized approach, while the assessment of HbA_1c_ levels at the beginning and end helped in understanding the program results in a resource-efficient and clinically effective manner.

### Strength and Limitations

The strength of this study is the analysis of data in real-world settings by using a commercial program. This preliminary analysis of the results was performed on participants with varied ages, treatment regimens, and baseline HbA_1c_ levels. This study was limited by the nonrandomized design, lack of control group, self-selection bias, and referral bias. This study takes into consideration the change in the glycemic outcome of the participants at the completion of 90 days of the program. Further studies using larger sample size, control groups, and longer durations will be required to understand the effectiveness of this digital diabetes care program. Control groups including healthy people without T2DM and people with T2DM without access to the digital therapeutics program can further help in understanding the efficacy of this program. The impact of such digital therapeutic programs on the psychological well-being and the physical fitness of participants will need to be further evaluated.

### Conclusion

Our study shows that participants with T2DM completing 90 days of the Fitterfly Diabetes CGM program showed a clinically significant improvement in glycemic control, as indicated by the significant reduction in HbA_1c_ levels, weight, and BMI. Moreover, the program helped in significantly improving glycemic control in people with different baseline HbA_1c_ values and in people on different treatment regimens of antidiabetic medications or not using any antidiabetic medications. During the 90 days of the program, a high level of participant engagement with the platform was observed. Our study also demonstrates a significant correlation between weight reduction and higher program participation metrics. Thus, the Fitterfly Diabetes CGM program can be an effective tool for improving glycemic control in people with T2DM by providing multidisciplinary care based on personalized glycemic responses.

## Abbreviations

CGM: continuous glucose monitoring
HbA_1c_: hemoglobin A1c
T2DM: type 2 diabetes mellitus
TAR: time above range
TBR: time below range
TIR: time in range

## Data Availability

The data sets generated during and analyzed during this study are not publicly available due to the contractual obligations of Fitterfly Healthtech Pvt Ltd, but deidentified data sets are available from the corresponding author on reasonable request.
